# Arterial transit artifact as a short-term prognostic indicator in acute ischemic stroke

**DOI:** 10.1186/s12883-024-03560-z

**Published:** 2024-02-09

**Authors:** Min Shan, Kaili Liu, Yi Ma, Qingxiu Zhang, Wenwei Yun, Min Zhang

**Affiliations:** 1 Department of Neurology, the Affiliated Changzhou Second People’s Hospital of Nanjing Medical University, Changzhou Second People’s Hospital, Changzhou Medical Center, Nanjing Medical University, No.29, Xinglong Lane, Tianning District, Changzhou, 213004 Jiangsu Province China; 2 Department of Radiology, the Affiliated Changzhou Second People’s Hospital of Nanjing Medical University, Changzhou Second People’s Hospital, Changzhou Medical Center, Nanjing Medical University, Changzhou, Jiangsu Province China; 3 Department of Neurology, Nanjing Drum Tower Hospital Affiliated to Medical School of Nanjing University, Nanjing, Jiangsu Province China

**Keywords:** Arterial spin labeling, Magnetic resonance imaging, Arterial transit artifact, Acute ischemic stroke, Stroke subtypes

## Abstract

**Background:**

Arterial transit artifact (ATA) observed on arterial spin labeling (ASL) was recently suggested to be associated with improved functional outcomes following acute ischemic stroke (AIS). AIS is a heterogeneous disease with diverse pathogenic mechanisms depending on the stroke subtype. This study aimed to investigate the association between ATA and 3-month functional outcomes in AIS patients according to etiology subtypes.

**Methods:**

Consecutive patients with AIS were included. All patients underwent ASL MRI with postlabeling delay (PLD) of 1.5 and 2.5 s. ATA was assessed from the ASL images of both PLDs. Stroke etiologic subtypes were determined according to the modified TOAST (Trial of ORG 10172 in Acute Stroke Treatment) classification. Short-term functional outcomes were evaluated using the 3-month modified Rankin scale (mRS). Log-binomial regression was applied to analyze the association between ATA and functional outcomes at 3 months after stroke.

**Results:**

Ninety-eight AIS patients (62.73 ± 13.05 years; 68 men) were finally included. ATA was detected in forty-six patients and most frequently seen in the large-artery atherosclerosis (LAA) subtype (35/46). The ATA group exhibited a lower percentage of patients with mRS > 2 compared to the group without ATA (36.5% vs. 19.6%; *P* < 0.001). ATA was independently associated with better 3-month clinical outcomes (adjusted risk ratio, 0.35[95% CI, 0.16—0.74]) in the multivariate log-binomial regression model. After stratification by TOAST subtypes, a significant association was found between ATA and better outcomes in the LAA subtype (adjusted risk ratio, 0.20[ 95% CI, 0.05—0.72]) but not in cardioembolism and small artery occlusion (SVO) subtype.

**Conclusion:**

ATA is associated with better outcomes at 3 months in patients with AIS, especially in the LAA subtype, but this association attenuated in the cardioembolism and SVO subtypes.

## Introduction

Arterial spin labeling (ASL) is a non-contrast magnetic resonance imaging (MRI) technique that assesses cerebral blood flow (CBF) quantitatively by magnetically labeling blood water [1]. Not only does ASL allow quantification of CBF, but it also enables visual evaluation of arterial transit artifact (ATA). ATA is identifiable as bright signals observed on ASL perfusion images in the vessels overlying the brain surface, indicating a delay in the arrival of labeled blood in the corresponding vascular territory [2]. ATA occurs when the arterial transit time (ATT) is longer than the postlabeling delay (PLD). It is recently suggested that ATA is a robust imaging marker of better prognosis in patients with ischemic stroke [3–5]. However, the evidence about the role of ATA in different ischemic stroke subtypes is limited. Acute ischemic stroke (AIS) is a major cause of disability and death, with variations in pathogenesis and perfusion compensatory mechanisms among different stroke subtypes [6]. The aim of this study was to explore the association between ATA and 3-month functional outcomes in AIS patients with different TOAST subtypes.

## Methods

### Patients

**Fig. 1:**
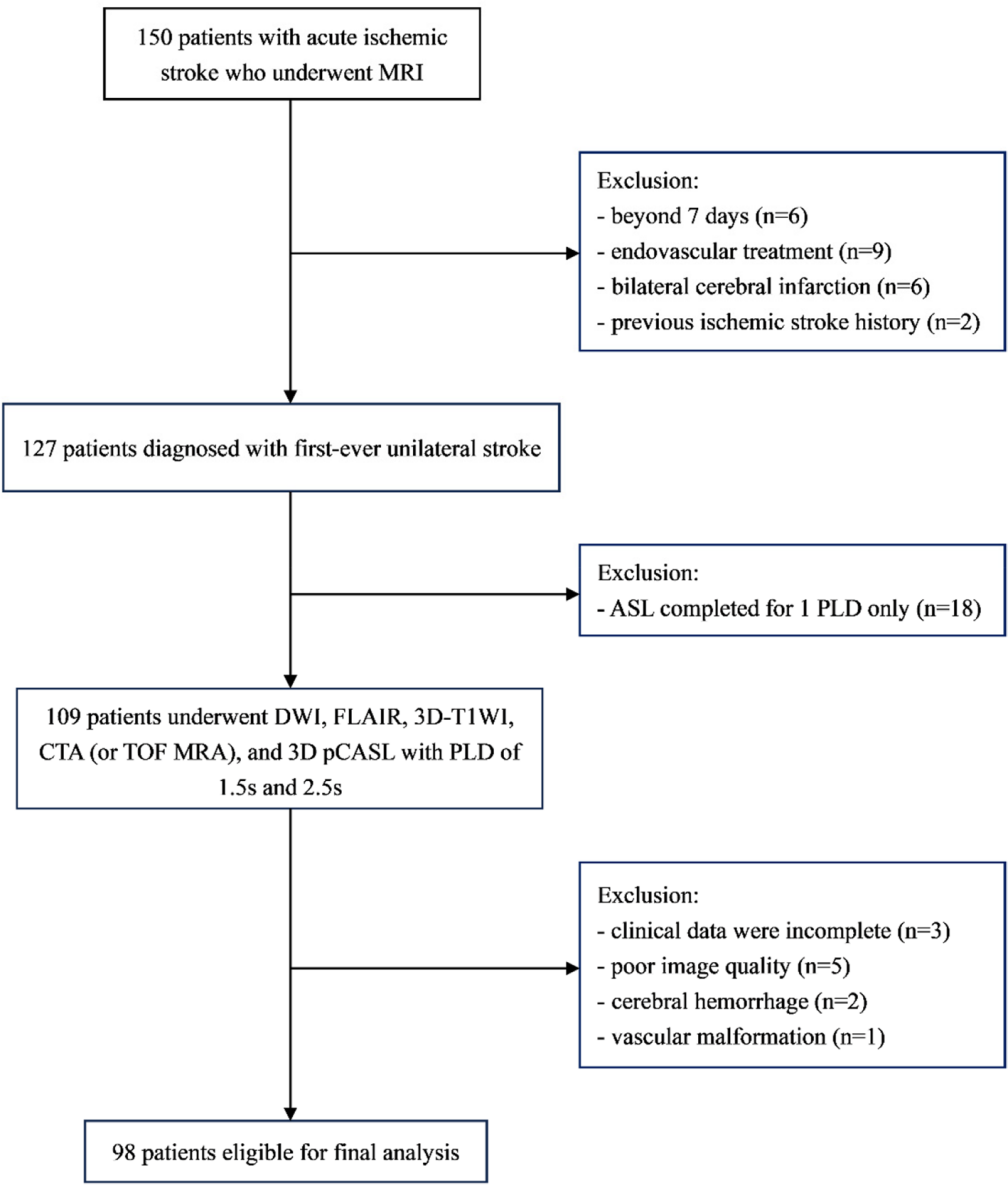
The flow chart of patient selection. ASL, arterial spin labeling; PLD, postlabeling delay; DWI, diffusion-weighted imaging; FLAIR, fluid attenuation inversion recovery; 3D-T1WI: 3-dimensional T1-weighted images; CTA, CT angiography; TOF MRA, time-of-flight MR angiography; 3D pCASL, 3-dimensional pseudo-continuous ASL

Consecutive patients diagnosed with AIS were enrolled from November 2020 to August 2022. All patients were diagnosed and hospitalized at the Department of Neurology, Changzhou Second People’s Hospital, Nanjing Medical University. A patient selection flow chart is shown in Fig. 1. Inclusion criteria were: (1) first-ever unilateral stroke within seven days, (2) age ≥ eighteen years, (3) technically adequate angiography (computed tomography angiography or magnetic resonance arteriography), (4) no thrombolysis or arterial thrombectomy was performed, and (5) informed consent was acquired from either the patients themselves or their families. By comprehensively analyzing the data and test results, stroke pathogenetic subtypes were determined as large-artery atherosclerosis (LAA), cardioembolism (CE), small-vessel occlusion (SVO) based on the modified TOAST criteria (Trial of ORG 10172 in Acute Stroke Treatment) [7]. Exclusion criteria involved as follows: (1) stroke of other clear etiology or unknown etiology (e.g., combined atrial fibrillation and LAA), (2) MRI contraindications, (3) combined with brain trauma, cerebral hemorrhage, tumor, vascular malformation, and other neurological diseases that can lead to deviation of results, and (4) uncertainty about the time of stroke onset. The institutional ethics committee of the Changzhou No.2 People's Hospital Affiliated to Nanjing Medical University (NO. 2018-KY032-01) approved the study. Patients with intravenous thrombolysis or thrombectomy were excluded to avoid possible hemodynamic changes and effects on prognosis. All enrolled patients completed MRI within 48 h after admission. National Institutes of Health Stroke Scale (NIHSS) at baseline was measured before any intervention. The Modified Rankin Scale (mRS) was assessed at 3 months after stroke over the phone or during a clinical visit. A mRS score of 0–2 at 3 months was considered as a favorable functional outcome, while an unfavorable outcome was characterized by a score of 3–5. We comprehensively collected demographic data, laboratory test results, and vascular risk factors of patients.

### Image acquisition and analysis

Imaging was acquired at 3.0 T Discovery 750W scanner (GE Healthcare, Waukesha, WI, USA). MR protocol includes diffusion-weighted imaging (DWI), T2 fluid attenuation inversion recovery (FLAIR), 3D-T1WI, CT angiography (CTA) or 3D time-of-flight MR angiography (TOF MRA), and 3D pseudo-continuous ASL (pCASL) with PLD of 1.5 s and 2.5 s.

ASL images were obtained with the following parameters: 36 sagittal slices; slice thickness = 4 mm; echo time = 10.7 ms; labeling duration = 1500 ms; repetition time = 4640 ms (PLD = 1.5 s) and 5335 ms (PLD = 2.5 s); field of view = 24 × 24 cm; number of excitation = 2 and background suppressed. 3D-T1WI images were obtained with the following parameters: TR = 7.5 ms, TE = 2.8 ms, inversion time (TI) = 450 ms, FA = 15°, FOV = 256 mm × 256 mm, matrix = 256 × 256, slice thickness = 1.0 mm and 156 sagittal slices. The T2 FLAIR sequences were obtained with the following parameters: TR = 12000 ms, TE = 123 ms, FA = 160°, TI = 2500 ms, matrix = 256 × 256, number of slices = 18, and slice thickness = 6.0 mm. DWI images were obtained with the following parameters: TR = 6000 ms, TE = 80 ms, FA = 90°, b = 1000 s/mm2, slice thickness = 6.0 mm, and number of slices = 18.

An investigator (Shan) preprocessed MRI data using SPM12 (http://www.fil.ion.ucl.ac.uk/spm/). Preprocessing steps entailed: (1) coregistration of ASL CBF maps to the 3D-T1WI; (2) segmentation of the 3D-T1WI into probabilistic masks for gray matter, white matter, and CSF, which were then normalized to the standardized Montreal Neurological Institute space; (3) resampling the normalized CBF maps to 3 mm × 3 mm × 3 mm isotropic voxel size; (4) the normalized CBF maps smoothed with a 5 mm isotropic Gaussian kernel. Mean CBF values of the affected and unaffected hemispheres were both calculated. Rest 1.8 tool based on Matlab was used for generation of cerebral hemisphere mask and extraction of CBF. Mean CBF values of the affected and unaffected hemispheres were both calculated.

Two experienced radiologists (Ma and Liu) independently and separately reviewed the ASL images and identified ATA. The stroke subtype was determined by two board-certified neurologists (Shan and Zhang) based on medical history and imaging data.

**Fig. 2:**
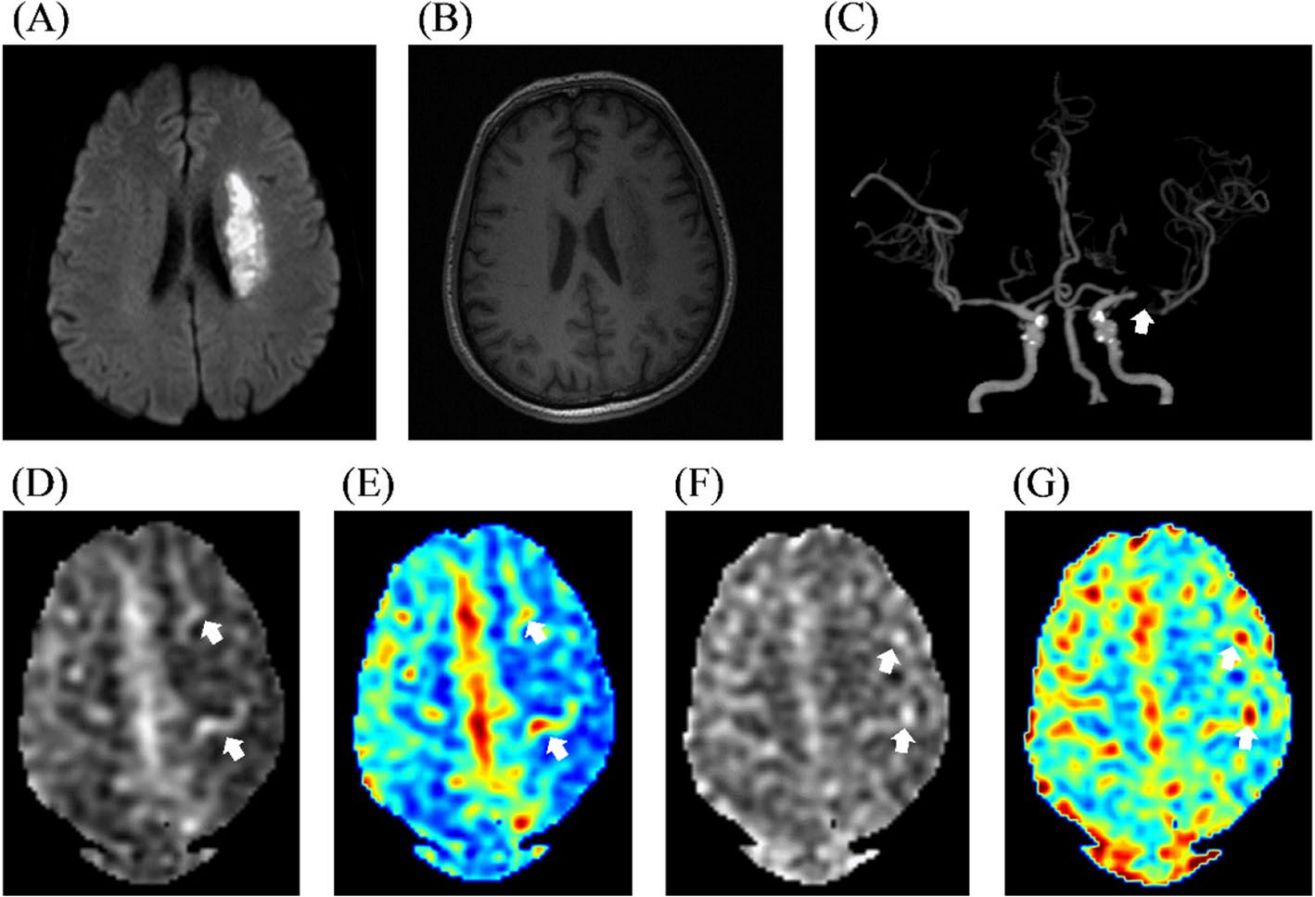
Imaging features of arterial transit artifact. **A **DWI showed infarction of the left basal ganglia and corona radiata. **B **Infarction was depicted on 3D-T1WI. **C **CTA showed occluded MCA and formation of collateral circulation distal to the occlusion. **D**, **E **ASL of 1.5 s PLD showed decreased CBF in the left MCA territory and visible arterial transit artifact. F&G, ASL of 2.5 s PLD showed decreased CBF in the left MCA territory and visible arterial transit artifact. DWI: diffusion-weighted imaging; 3D-T1WI: 3-dimensional T1-weighted images; CTA, CT angiography; MCA, left middle cerebral artery; ASL, arterial spin labeling; PLD, postlabeling delay; CBF, cerebral blood flow

ATA was defined as serpiginous or punctate high intensity surrounding the hypoperfused infarct area on CBF images (Fig. 2). It is considered as ‘ATA present’ no matter on which PLD ASL perfusion images ATA is found.

3D Slicer, version 4.11 was used to segment and reconstruct the infarct according to the signal difference between the lesion and the normal regions. The volume of the infarct was calculated in a semi-automatic manner, with each slice being analyzed pixel by pixel.

### Statistical analysis

Statistical analyses were performed using Stata, version 17.0 (StataCorp LP, College Station, Texas). Normality for continuous variables was examined using Skewness–Kurtosis test. For continuous variables, mean and standard deviation were used to present normally distributed data, while median and interquartile range (IQR) were used for skewed data. To compare continuous variables, independent-samples two-tailed t test or Mann–Whitney U test were conducted. Categorical variables were analyzed using χ^2^ test. The associations of ATA with outcomes were explored with log-binomial regression models. We used the χ^2^ test to compare the differences between TOAST subtype groups. Cohen's kappa coefficient was utilized to evaluate the level of agreement between two observers. *P* value of < 0.05 was considered statistically significant.

## Results

### Characteristics of patients

Of one hundred and fifty patients who were diagnosed with AIS in the study period, Six patients were excluded due to stroke onset of more than 7 days; nine patients were excluded because of a history of endovascular treatment; two patients were excluded because of a history of previous ischemic stroke; six patients were excluded due to bilateral cerebral infarction; eighteen patients were excluded because ASL was completed for only one PLD; three patients were excluded because of insufficient clinical data; moreover, five patients with poor image quality, two with cerebral hemorrhage and one with vascular malformation were excluded.

Ninety-eight patients (mean age 62.73 ± 13.05 years; 68 men) were eventually included in this study. Among them, 67 patients underwent CTA only, 27 patients underwent MRA only, and 4 patients underwent both CTA and MRA. The characteristics of patients are shown in Table 1. Of the 98 patients, 44 (44.9%) were LAA-subtype, 17 (17.3%) were CE-subtype, and 37 (37.8%) were SVO-subtype. The patients had a median admission NIHSS score of 3 (IQR, 2–4), and the median infarct volume on DWI was 1.3 mL (IQR, 0.6–2.1 mL). 54 patients received single antiplatelet therapy and 44 received dual antiplatelet therapy. ATA was observed in 46 patients. 28 patients (28.6%) had a mRS score above 2 at the 3-month follow-up.

**Table 1:** Patient characteristics according to presence or absence of arterial transit artifact

	All patients (*n* = 98)	ATA present (*n* = 46)	ATA absent (n = 52)	*P* value
Age, y	62.7 (13.1)	62.7 (12.9)	62.8 (13.3)	0.966
Sex, male	68 (69.4)	31 (67.4)	37 (71.2)	0.687
History				
Hypertension	78 (79.6)	38 (82.6)	40 (76.9)	0.486
Diabetes mellitus	28 (28.6)	19 (41.3)	9 (17.3)	0.009^*^
Obesity	13 (13.3)	8 (17.4)	5 (9.6)	0.257
Smoking	40 (40.8)	21 (45.7)	19 (36.5)	0.360
Laboratory data				
TG, mmol/L	1.475 (1.19, 2.57)	1.415 (1.15, 2.32)	1.61 (1.205, 2.705)	0.906
TC, mmol/L	3.965 (3.25, 4.71)	4.06 (3.27, 4.86)	3.93 (3.17, 4.66)	0.584
HDL-C, mmol/L	0.985 (0.86, 1.18)	1.02 (0.86, 1.20)	0.965 (0.84, 1.15)	0.424
LDL-C, mmol/L	2.425 (1.92, 2.96)	2.445 (2.01, 2.96)	2.385 (1.835, 2.985)	0.436
FBG, mmol/L	5.12 (4.56, 5.9)	5.395 (4.67, 6.83)	4.87 (4.465, 5.655)	0.029^*^
HbA1c, %	6 (5.6, 6.8)	6.2 (5.7, 7.4)	5.85 (5.6, 6.3)	0.064
BUN, mmol/L	5 (4.1, 6.3)	4.95 (4.2, 5.5)	5.2 (4.05, 6.6)	0.380
UA, µmol/L	316.1 (82.6)	314.0 (83.73)	317.97 (82.31)	0.816
Cr, µmol/L	72.5 (60, 82)	69.5 (59, 84)	73.5 (64, 81)	0.669
HCY, mol/L	10.9 (8.2, 13.6)	10.6 (7.7, 15.5)	11.25 (8.7, 13.3)	0.836
NIHSS baseline	3 (2, 4)	3 (2, 4)	2 (2, 3.5)	0.163
Infarct volume, mL	1.3 (0.6, 2.1)	1.65 (0.1, 2.7)	1 (0.5, 1.6)	< 0.001^*^
TOAST subtype				< 0.001^*^
LAA	44 (44.9)	35 (76.1)	9 (17.3)	
CE	17 (17.3)	5 (10.9)	12 (23.1)	
SVO	37 (37.8)	6 (13.0)	31 (59.6)	
In hospital treatment				0.888
Single antiplatelet	54 (55.1)	25 (54.3)	29 (55.8)	
Dual antiplatelet	44 (44.9)	21 (45.7)	23 (44.2)	
Ipsilateral hemisphere				
CBF_PLD = 1.5 s_, mL/(100 g‧min)	36.39 (32.33, 40.48)	36.39 (31.81, 40.48)	36.03 (33.28, 40.45)	0.458
CBF_PLD = 2.5 s_, mL/(100 g‧min)	41.53 (4.63)	41.96 (4.89)	41.14 (4.40)	0.384
CBF difference_ipsi_, mL/(100 g‧min)	4.96 (3.52)	5.40 (2.70)	3.84 (3.34)	0.013^*^
Contralateral hemisphere				
CBF_PLD = 1.5 s_, mL/(100 g‧min)	36.71 (34.47, 40.28)	36.58 (33.51, 41.61)	37.27 (34.58, 39.82)	0.749
CBF_PLD = 2.5 s_, mL/(100 g‧min)	41.91 (39.44, 46.99)	41.84 (38.71, 47.13)	42.02 (39.68, 46.33)	0.918
CBF difference_contra_, mL/(100 g‧min)	4.58 (3.14)	5.36 (2.83)	4.62 (4.02)	0.301
mRS > 2	28 (28.6)	9 (19.6)	19 (36.5)	< 0.001^*^

Data presented as n (%), mean ± standard deviation, or median (interquartile range)

*ATA* arterial transit artifact, *TG* triglyceride, *TC* total cholesterol, *HDL-C* high-density lipoprotein cholesterol, *LDL-C* low-density lipoprotein cholesterol, *FBG* fasting blood glucose, *HbA1c* glycated hemoglobin, *BUN* blood urea nitrogen, *UA* uric acid, *Cr* creatinine, *HCY* homocysteine, *NIHSS* National Institutes of Health Stroke Scale, *TOAST* Trial of ORG 10172 in Acute Stroke Treatment, *LAA* large-artery atherosclerosis, *CE* cardioembolism, *SVO* small-vessel occlusion, *PLD* postlabelling delay, *CBF* cerebral blood flow, *mRS* modified Rankin Scale

^*^P < 0.05

### Comparison between patients with ATA and without ATA

**Fig. 3:**
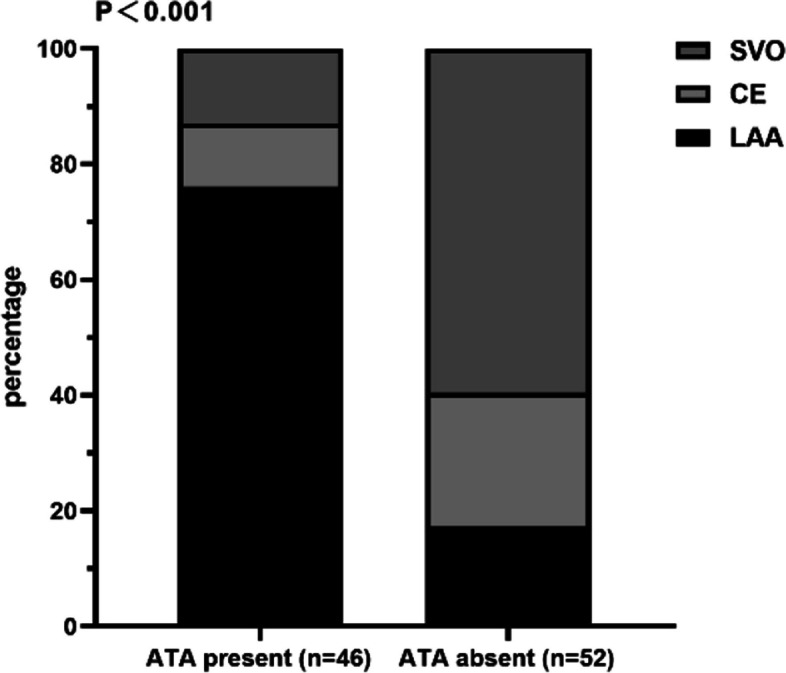
Toast subtypes composition comparison between patients with and without ATA. TOAST, Trial of ORG 10172 in Acute Stroke Treatment; ATA, arterial transit artifact; LAA, large-artery atherosclerosis; CE, cardioembolism; SVO, small-vessel occlusion

The reliability of inter-observer assessment for ATA was found to be excellent (1.5 s PLD: κ = 0.92, 2.5 s PLD: κ = 0.95). Differences were agreed upon after a joint review. Patients who were present with ATA (*n* = 46) and without ATA (*n* = 52) had similar baseline ages (62.7 vs. 62.8 years; *P* = 0.966). The presence of ATA was more frequent in patients with diabetes mellitus (19/46; 41.3%) than in patients without diabetes mellitus (9/52; 17.3%) (*P* = 0.009). Compared with patients without ATA, those with ATA had higher fasting blood glucose (*P* = 0.029) and larger DWI lesion size (*P* < 0.001). ATA was found more in patients diagnosed with LAA (*P* < 0.001; Fig. 3). In particular, the difference of CBF between 1.5 s and 2.5 s PLD in the affected hemisphere was higher in patients with ATA (*P* = 0.013). A lower proportion of patients with ATA had an mRS > 2 compared with those without ATA (36.5% vs. 19.6%; *P* < 0.001), as presented in Table 1.

### Association between ATA and 3-month functional outcomes

In the univariate analysis, NIHSS at baseline, lesion volume, and ATA were identified as significant variables for outcome prediction (Table 2). The multivariable analysis revealed that ATA remained significantly associated with the favorable 3-month clinical outcome after adjusting for other significant influencing factors (adjusted RR, 0.35 [95% CI, 0.16—0.74]).

**Table 2:** Univariate and multivariable analyses (Log-binomial regression) analyses for outcomes (mRS > 2) while controlling for potential confounders

	Univariate analysis (Unadjusted)		Multivariable analysis^a^ (Adjusted)	
	Risk ratios (95% CI)	*P* Value	Risk ratios (95% CI)	*P* Value
Age	1.00 (0.98—1.03)	0.702	—	—
Sex	1.05 (0.52—2.12)	0.897	—	—
Hypertension	0.90 (0.42—1.92)	0.781	—	—
Diabetes mellitus	0.88 (0.42—1.84)	0.724	—	—
Obesity	0.81 (0.29—2.33)	0.706	—	—
Smoking	0.73 (0.36—1.45)	0.362	—	—
FBG	0.94 (0.78 – 1.14)	0.550	—	—
NIHSS baseline	1.25 (1.13—1.38)	< 0.001^*^	1.17 (1.04—1.32)	0.010^*^
Infarct volume	1.17 (1.01—1.36)	0.037^*^	1.25 (1.06—1.47)	0.007^*^
TOAST subtype^b^				
LAA	0.76 (0.37—1.60)	0.475	—	—
CE	1.19 (0.53 – 2.67)	0.679	—	—
SVO	1.00	—	—	—
In hospital treatment	0.52 (0.25—1.07)	0.075	—	—
ATA	0.48 (0.23 – 0.98)	0.046^*^	0.35 (0.16—0.74)	0.006^*^

*mRS* modified Rankin Scale, *FBG* fasting blood glucose, *NIHSS* National Institutes of Health Stroke Scale, *TOAST* Trial of ORG 10172 in Acute Stroke Treatment, *LAA* large-artery atherosclerosis, *CE* cardioembolism, *SVO* small-vessel occlusion, *ATA* arterial transit artifact

^a^Multivariable logistic regression model for mRS > 2, adjusting for potential confounders (NIHSS at baseline and infarct volume)

^b^SVO is used as a reference

^*^P < 0.05

All enrolled patients were divided into 3 groups according to the modified TOAST criteria. In the unadjusted model, ATA appeared to be linked to better 3-month functional outcomes in the LAA patients (RR, 0.26 [ 95% CI, 0.09–0.70]) (Table 3). After adjustment for baseline covariates, ATA remained significantly associated with better outcomes in the LAA subtype (adjusted RR, 0.20 [95% CI, 0.05—0.72). However, neither the unadjusted nor adjusted analysis showed that ATA increased the possibility of better outcomes in the CE and SVO subtypes.

**Table 3:** The association between the arterial transit artifact and 3-month outcomes stratified by TOAST subtypes

			Risk ratios (95% CI)	
	n	3-month mRS > 2	Unadjusted	Adjusted^a^
LAA	44			
ATA present	35	5 (14.29)	0.26 (0.09- 0.70)	0.20 (0.05—0.72)
ATA absent	9	5 (55.56)	Reference	Reference
CE	17			
ATA present	5	2 (40.00)	1.20 (0.31—4.58)	3.55 (0.43—29.27)
ATA absent	12	4 (33.33)	Reference	Reference
SVO	37			
ATA present	6	1 (16.67)	0.52 (0.08—3.32)	0.51 (0.08—3.57)
ATA absent	31	10 (32.26)	Reference	Reference

*ATA* arterial transit artifact, *TOAST* Trial of ORG 10172 in Acute Stroke Treatment, *LAA* large-artery atherosclerosis, *CE* cardioembolism, *SVO* small-vessel occlusion, *mRS* modified Rankin Scale

^a^Adjusted for potential confounders (NIHSS at baseline and infarct volume)

### Comparison of ATA occurrence between patients stratified by stroke etiologic subtypes

There was a significant difference in the incidence of ATA among the three subtype groups, with the LAA group exhibiting a higher occurrence rate. ATA appeared more frequently in 1.5 s PLD than in 2.5 s PLD. Data on the occurrence of ATA is shown in Table 4.

**Table 4:** Prevalence of ATA stratified by TOAST subtypes

	LAA (*n* = 44)	CE (*n* = 17)	SVO (*n* = 37)	*P* value
ATA	35	5	6	< 0.001^*^
ATA_PLD = 1.5 s_	33	4	6	< 0.001^*^
ATA_PLD = 2.5 s_	17	3	4	0.012^*^

*TOAST* Trial of ORG 10172 in Acute Stroke Treatment, *ATA* arterial transit artifact, *LAA* large-artery atherosclerosis, *CE* cardioembolism, *SVO* small-vessel occlusion

^*^P < 0.05

## Discussion

In this retrospective study, we found that ATA on ASL images was associated with more favorable 3-month clinical outcomes following AIS. After stratifying patients by TOAST subtypes, a significant association between ATA and better 3-month functional outcomes in LAA patients was found. However, our findings indicated that this relationship was not statistically significant in CE and SVO subtypes.

The presence of ATA is determined by the competition between PLD and ATT [2]. The PLD allows the movement of labeled water from the neck to the capillary bed of the brain [8]. In cases where the PLD is insufficient for the labeled blood to reach the capillary bed (shorter PLD vs. longer ATT), labeled blood may be retained in the supplying arteries during imaging [9], leading to high ASL signal intensity in the affected vascular territory, which is referred to as ATA. In addition, we found that ATA appeared more frequently in 1.5 s PLD than in 2.5 s PLD. The selection of PLD may affect ATA frequency.

In the past decades, ATA was misinterpreted as a confounder affecting the analysis of ASL images. However, recent studies have found that the presence of ATA was associated with better functional outcomes of AIS patients. Consistent with these studies, our study demonstrated the positive impact of ATA on the recovery of neurofunction in AIS patients. We found a significant correlation between the presence of ATA and favorable outcomes of AIS in univariate analyses. The prognostic significance of ATA for AIS remained statistically significant after adjustment for potential confounders. ATA has been reported as an indicator of improved collateral flows [10, 11]. The delayed perfusion it induces may prevent the progression of infarction, thereby improving the prognosis [12, 13]. The presence of ATA can serve as a valuable diagnostic indicator, as it is sensitive to arterial arrival time and can help identify patients with a higher likelihood of achieving positive outcomes.

We justly found the independent prediction of ATA for ischemic stroke outcomes in patients with the LAA subtype, rather than the others. Ischemic stroke is a heterogeneous disease with varying pathogenesis. In LAA patients, the presence of ATA may imply the recruitment of collaterals through leptomeningeal vessels, the circle of Willis, or other large arteries surrounding the obstruction to ensure the perfusion level, which is beneficial to the prognosis [14]. The reason for the lack of a similar correlation for cardioembolic stroke compared with LAA may lie in the fact that stroke caused by cardiac factors tend to involve rapid occlusion and a high thrombus load, making it difficult to form collateral circulation [15]. In addition, the number of patients with CE subtype in this study was relatively small. The relationship between ATA and functional outcomes of CE patients may change if the sample size is increased.

Our findings suggest that ATA was found mostly in LAA-subtype patients. This can be for several reasons. Firstly, patients with large artery stenosis or occlusion due to atherosclerosis often experience partial or complete obstruction of rapid antegrade flow [16]. Additionally, it takes more time for blood to arrive via collateral pathways. Therefore, ATA is more commonly detected in LAA patients. At the same time, small arteries such as penetrating arterioles lack anastomoses. Lacunar infarcts due to small vessel occlusion usually exhibit unimpeded rapid antegrade blood flow. Previous studies have reported a high occurrence of ATA in stenotic or occlusive diseases [17–19], suggesting that ATA may be meaningful for screening patients with potential high-grade large artery lesion and facilitating targeted intervention for these individuals.

Our study also found that the difference in CBF between 2.5 s and 1.5 s PLD in the affected cerebral hemisphere was higher in the group with ATA compared to the group without ATA. Perfusion information with different PLD can be effectively captured by ASL. At the PLD of 1.5 s, CBF primarily reflects perfusion of the collateral flow that reaches the downstream territory more rapidly. On the other hand, the CBF at 2.5 s PLD reflects tortuous retrograde collateral perfusion and some possible slow antegrade flow [20]. The 2.5 s PLD allows more ample time for blood flow to reach the blood supply area, potentially containing more collateral compensatory blood flow. Previous studies have suggested that the CBF difference between the two PLDs may partially reflect the collateral circulation contribution [21].

To date, no studies were done to investigate the role of ATA in different subtypes of AIS. As the first study on ATA, TOAST subtypes, and short-term outcomes, the results underscore the prognostic significance of ATA and the necessity of TOAST classification during investigation of prognostic markers of AIS. Previous research suggested that ATA improves functional outcomes mainly because it represents an improvement in collateral circulation [4]. Our findings further support the possibility of the collateral compensation mechanism and provide evidence for clarifying the role of ATA in stroke.

Additionally, we found that ATA was more prevalent in patients with diabetes mellitus. Diabetes mellitus is one of the most important risk factors for atherosclerosis [22]. Higher incidence of diabetes was associated with higher incidence of both ATA and LAA. The increased prevalence of ATA in diabetes patients may be mediated by the higher prevalence of diabetes in atherosclerotic patients.

ASL is a non-invasive MRI method that magnetically labels blood water [23]. Unlike CT perfusion-weighted imaging or MRI contrast-enhanced methods, which have been commonly used in previous neuroimaging studies, ASL enables quantitative perfusion measurement without the need for exogenous contrast agents [24]. Prior studies have demonstrated that calculated CBF values obtained from ASL images in AIS patients are consistent with those derived from dynamic susceptibility contrast (DSC) perfusion images [25]. Calculating CBF from ASL needs some dedicated software and postprocessing steps, whereas ATA can be identified through straightforward visual inspection of ASL images, making it a potentially more extensively used imaging marker in clinical practice.

This study had several limitations. Firstly, only monocentric participants with relatively small sample size were enrolled, which in part restricts the clinical generalizability of our findings. Further research requires external validation and more data. Secondly, we defined the presence of ATA as either appearing on 1.5 s or 2.5 s PLD ASL images. The utilization of a PLD of 1.5 s might have contributed to a higher incidence of ATA, as ATA occurs when the PLD is shorter than the ATT. Thirdly, further evaluation of specific lesioned vessels and the degree of arterial occlusion was not conducted in patients with LAA. Finally, although we controlled the time between admission and MR examination of the enrolled patients, we could not manage to completely avoid the effect of this period of time on ATA formation. Future studies should try to control for a consistent time between the onset and the time the patient underwent the examination, or include the examination time window in the analysis.

## Conclusion

ATA obtained from ASL is a valuable imaging marker for predicting short-term functional outcomes in patients with AIS, particularly those with LAA subtype. ATA was meaningful for screening patients with potential high-grade large artery lesion. ATA may assist in guiding the prognosis and management of AIS, especially for patients who are unable to undergo contrast-enhanced radiologic examinations.

## Data Availability

The data that support the findings of this study are available on request from the corresponding author, Min Zhang, upon reasonable request.
